# Electronic Properties of Triangle Molybdenum Disulfide (MoS_2_) Clusters with Different Sizes and Edges

**DOI:** 10.3390/molecules26041157

**Published:** 2021-02-22

**Authors:** Songsong Wang, Changliang Han, Liuqi Ye, Guiling Zhang, Yangyang Hu, Weiqi Li, Yongyuan Jiang

**Affiliations:** 1 School of Physics, Harbin Institute of Technology, Harbin 150001, China; sswang@hit.edu.cn; 2 Key Lab of Micro-Optics and Photonic Technology of Heilongjiang Province, Harbin 150001, China; 3 School of Mechanical & Power Engineering, Harbin University of Science and Technology, Harbin 150080, China; hanchangliang@hrbust.edu.cn; 4 Key Laboratory of Green Chemical Technology of College of Heilongjiang Province, College of Chemical and Environmental Engineering, Harbin University of Science and Technology, Harbin 150080, China; yeliuqi99@163.com (L.Y.); guiling-002@163.com (G.Z.); 5 State Key Laboratory of Intense Pulsed Radiation Simulation and Effect, Xi’an 710024, China

**Keywords:** molybdenum disulfide clusters, electronic properties, quantum chemistry, nanomaterials

## Abstract

The electronic structures and transition properties of three types of triangle MoS_2_ clusters, **A** (Mo edge passivated with two S atoms), **B** (Mo edge passivated with one S atom), and **C** (S edge) have been explored using quantum chemistry methods. The highest occupied molecular orbital (HOMO)–lowest unoccupied molecular orbital (LUMO) gap of B and C is larger than that of A, due to the absence of the dangling of edge S atoms. The frontier orbitals (FMOs) of A can be divided into two categories, edge states from S_3*p*_ at the edge and hybrid states of Mo_4*d*_ and S_3*p*_ covering the whole cluster. Due to edge/corner states appearing in the FMOs of triangle MoS_2_ clusters, their absorption spectra show unique characteristics along with the edge structure and size.

## 1. Introduction

Since the successful isolation of graphene [1,2], atomic layer-thick two-dimensional (2D) nanomaterials have attracted a great deal of attention due to their excellent mechanical flexibility and optical transparency, as well as their ultrahigh specific surface, which are related to their strong in-plane covalent bond and atomic thickness [3,4,5,6,7]. Beyond graphene, single-layer transition-metal dichalcogenides (TMDs) have been receiving increasing interest. For example, the structural and physical properties of the MoS_2_ monolayer and bulk have been extensively studied both theoretically and experimentally. The monolayer of MoS_2_ consists of a layer of Mo atoms sandwiched between two layers of S atoms, forming a trilayer. The absence of inversion symmetry and dominant *d*-electron interactions of the heavy transition metal atom Mo, endow the monolayer (MoS_2_) with intriguing physical properties that are not found in *sp*-bonded nanomaterials [8,9,10,11].

In recent years, an increasing amount of effort has been devoted to 2D materials with ultrasmall sizes, due to increasing demand for miniaturizing photonic and optoelectronic devices. For MoS_2_, from the three-dimensional (3D) bulk structure to the 2D monolayer, the band gap transforms from an indirect band gap (∼1.2 eV) to a direct band gap (∼1.8 eV, in the visible frequency range) [12,13]. Compared with their 2D and one dimensional (1D) counterparts, zero dimensional (0D) MoS_2_ clusters possess tunable energy levels, more active edges, and larger surface-area-to-volume ratios, which give them fascinating properties. This makes them promising candidates for application in fields such as optoelectronics [14,15], electrochemical technology [16,17], biology [18], catalysis [19] and so forth [20,21,22]. Jaramillo et al. [23] pointed out that 2D TMDs are hydrogen evolution reaction (HER) active because of their highly active edges, which makes them potential candidates for electrocatalytic hydrogen evolution. Yin et al. [24] pointed out that a strong second-harmonic generation (SHG) can be observed at the edges and corners of MoS_2_ clusters, implying the important role that edges and corner states play in determining the properties of the 0D structures. However, the detailed transition natures are ambiguous. In order to shed light on the essence of the various applications and obtain full use of the MoS_2_ clusters, it is crucial to gain a comprehensive understanding of the electronic structures and their transition properties.

It is well known that when the physical size of the material is comparable to or smaller than the Bohr radius, the excitons are confined in all three dimensions, so there exists a 3D quantum confinement effect. Superimposed on the intrinsic size-dependent electronic structure, one thus has the effect of edges, corners, atomic vacancies and so forth, all these are likely to induce additional local effects. As a result, the electronic properties become increasingly sensitive not only to sizes, but to the shapes, edge atomic structures, and compositions [25,26,27]. Despite the breakthroughs in preparation of MoS_2_ clusters over the years, mainly including top-down [28,29,30,31,32,33] and bottom-up methods [34,35,36], a cost-effective yet efficient technique for the high yield production of MoS_2_ clusters with desirable sizes remains a challenge. Consequently, it is still difficult to provide a specific illustration about the properties of MoS_2_ clusters experimentally. Aiming to obtain the whole picture in relation to MoS_2_ clusters and to clarify the relationships between structures and properties, the density-of-states (DOS), frontier molecular orbitals (FMOs), electronic absorption spectra, and electron-hole characteristics of a series of triangular MoS_2_ clusters with different sizes and edges will be explored using quantum chemistry methods in the present paper. This will be of great significance not only in fundamental physics exploration but also in device applications.

## 2. Models and Computational Details

**Figure 1:**
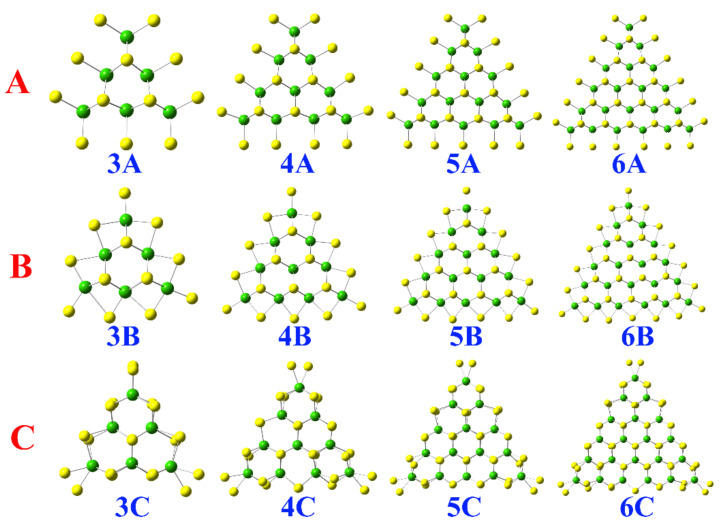
Top view of the triangle MoS_2_ clusters with different sizes and edges. The Mo and S atoms are represented by green and yellow spheres, respectively.

Based on different edge structures, there are mainly three types of triangle MoS_2_ clusters, as shown in Figure 1. **A** and **B** represent the Mo-terminated (commonly referred as Mo-edge) triangle MoS_2_ clusters, while **C** is the S-terminated cluster (commonly referred as S-edge). Considering that a bare Mo-edge is not stable, it is often passivated by one or two S atoms per edge-Mo atom. In **A**, each edge-Mo atom is passivated with two S atoms forming an S-dimer normal to the basal plane, usually called a Mo edge, with 100% S coverage.

In the case of **B**, two adjacent edge-Mo atoms share one S atom forming an S-monomer parallel to the basal plane, usually called a Mo edge, with 50% S coverage. Consequently, the S atoms of **C** dimerize along the direction normal to the basal plane, often called a S edge, with 100% coverage. By changing the number of the edge-Mo atoms n (*n* = 3, 4, 5, and 6) in **A**, **B**, and **C**, a series of different triangle MoS_2_ clusters with different sizes and edges could be gained. In the following discussions, **A** refers to four triangle MoS_2_ clusters with the same edges but different sizes, including **3A**, **4A**, **5A**, and **6A**, as **B** and **C**.

The density functional theory (DFT) based on Becke’s three-parameter hybrid exchange combined with the Lee–Yang–Parr correlation (B3LYP) [37,38] and the LANL2DZ basis set were adopted to optimize the geometries of the triangular MoS_2_ clusters. With both the merits of B3LYP and long-range corrected properties, CAM-B3LYP is able to predict molecular charge-transfer spectra properly due to the improved description of the long-range exchange interactions [39,40,41,42]. It is employed to evaluate the electron excitations with the LANL2DZ basis set in the present work.

The DFT calculations were performed using the Gaussian 09 program [43]. The data of DOS, electronic absorption spectra, and electron-hole distributions were obtained with the Multiwfn package [44].

## 3. Results and Discussion

In order to indicate the stability of **A**, **B**, and **C**, the formation energy was calculated according to the formula: *E*_formation_ = *E*_cluster_ (Mo_m_S_n_) − m*E* (Mo) − n*E* (S), where *E*_cluster_ (Mo_m_S_n_), *E* (Mo), and *E* (S) denote the energy of the cluster having m Mo atoms and n S atoms, the energy of a single Mo atom, and the energy of a single S atom, respectively. The calculated *E*_formation_ values of **A**, **B**, and **C** are listed in Table 1, Table 2 and Table 3, respectively. The negative *E*_formation_ values imply that the clusters are stable. The *E*_formation_ decrease with increasing the sizes of the cluster, suggesting that the larger the cluster, the more energy is released. 

**Figure 2:**
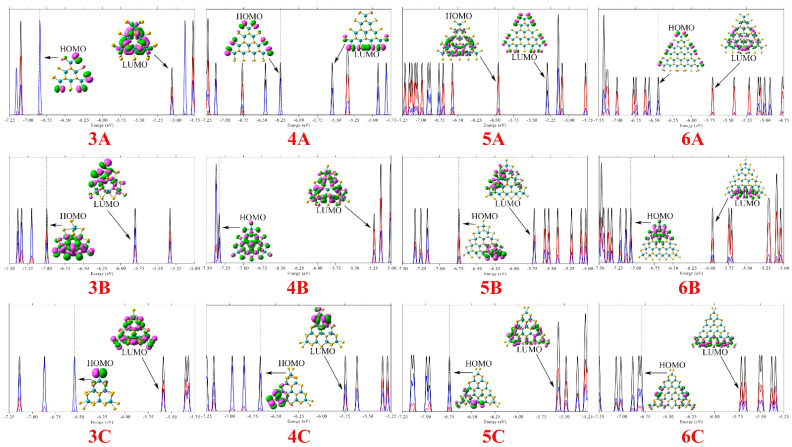
The density-of-states (DOS) map, as well as HOMO and LUMO distributions of **A**, **B**, and **C**. The discrete represents the MOs, the black, blue, and red curves represent the total DOS, partial DOS of S_3*p*_, and partial DOS of Mo_4*d*_, respectively.

For the three different classes of clusters, their highest occupied molecular orbital (HOMO) and lowest unoccupied molecular orbital (LUMO) energies and their energy gaps, as well as the orbital compositions of HOMO and LUMO are listed in Table 1, Table 2 and Table 3, respectively. The DOS is shown in Figure 2.

For these ultrasmall MoS_2_ clusters, the band gap does not decrease monotonically with size as is the case for graphite flakes, which might be attributed the deformation of structures due to large the ratio of number of edge atoms to core atoms in ultrasmall clusters. The energy of HOMO (*E*_H_) in **3A** is −5.06 eV, while that of LUMO (*E*_L_) is −6.84 eV, resulting in a 1.78 eV energy gap (*E*_gap_). The *E*_gap_ value of **4A**, **5A**, and **6A** is 0.70, 0.66, and 0.73 eV, respectively, far smaller than that of **3A**. For the Mo-terminated triangle MoS_2_ clusters with 100% S coverage, some FMOs contributed from S_3*p*_ at the edge exhibit evident edge states, and the other FMOs exhibit hybrid states of Mo_4*d*_ and S_3*p*_ whose charge distribution covers the whole cluster. By comparing the electronic structures of three classes of clusters, it can be seen that the energy gap of the ultrasmall MoS_2_ clusters is dependent on the edge structure, which is similar to graphite flake. For Mo-terminated triangle MoS_2_ clusters with 50% S coverage (**B**) and S-terminated triangle MoS_2_ clusters (**C**), their S atoms at the edge of the cluster are shared by two adjacent Mo atoms. In contrast to the **A** system, the absence of dangling edge S atoms in the two class clusters increases their HOMO–LUMO gap and evident edge/corner states can be observed in the FMOs of these clusters. For **B**, all the FMOs comes from the hybridization between Mo_4*d*_ and S_3*p*_. While the occupied FMOs mainly comes from the S_3*p*_, and the unoccupied FMOs from the hybridization between Mo_4*d*_ and S_3*p*_ in the case of **C**. 

**Figure 3:**
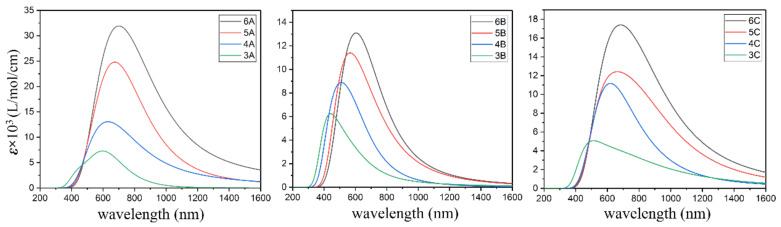
Electronic absorption spectra with full width at half maximum (FWHM) 0.26 of **A**, **B**, and **C**.

**Figure 4:**
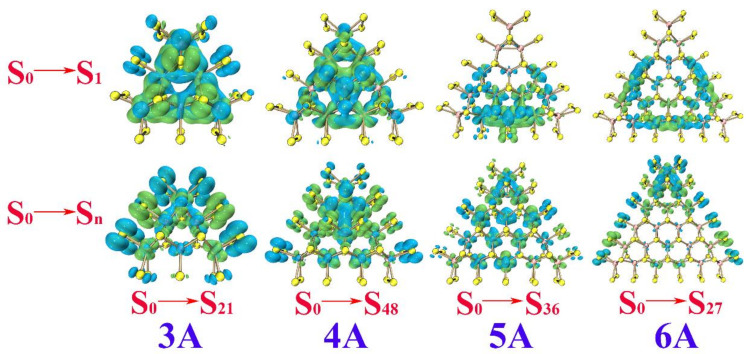
Electron (green) and hole (blue) distributions with isosurface 0.0006 for **3A**, **4A**, **5A** and **6A**.

**Figure 5:**
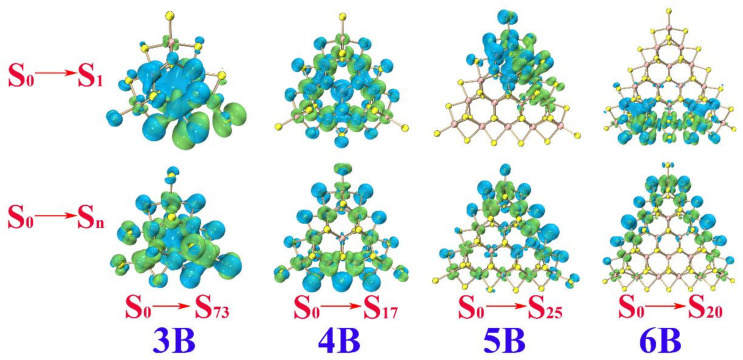
Electron (green) and hole (blue) distributions with isosurface 0.0006 for **3B**, **4B**, **5B** and **6B**.

**Figure 6:**
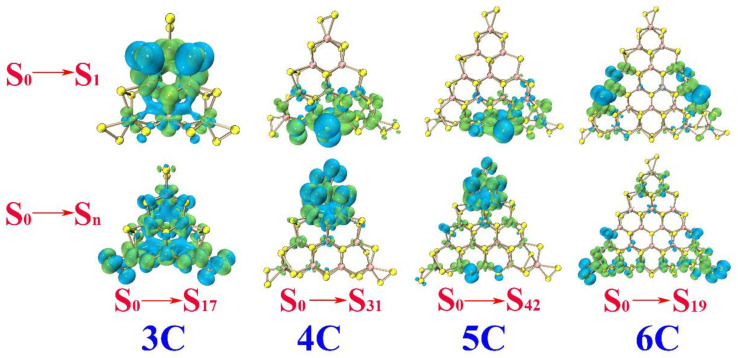
Electron (green) and hole (blue) distributions with isosurface 0.0006 for **3C**, **4C**, **5C** and **6C**.

The electronic absorption spectra of **A**, **B**, and **C** and their corresponding electron-hole distributions where the process of single-electron excitation is described as “an electron goes to the electron from the hole” are given in Figure 3, Figure 4, Figure 5 and Figure 6, respectively. Moreover, the percentage of Mo_4*d*_ and S_3*p*_ in the electron and hole are provided in Table 4, Table 5 and Table 6, respectively. The maximum absorption shows a red shift along with increasing size of the clusters in the **A**, **B** and **C** systems. For **3A**, **4A**, **5A**, and **6A**, the maximum absorption is ~623.61, ~591.50, ~670.54, and 796.87 nm, respectively. From Table 4, the percentage of S_3*p*_ is larger than that of Mo_4*d*_ in both the electron and hole in **3A**, Mo_4*d*_ takes the larger proportion in the electron and the hole in 4**A** and **5A**. The S_3*p*_ and Mo_4*d*_ make almost the same contribution to the electron and the hole in 6**A**. Combined with the data in Figure 4, there are two types of electron transitions: one is a *d*-*d* transition of the Mo atoms, and the other occurs at the S atoms along the edges, which is associated with the edge states.

The maximum absorption is ~422.32, ~609.91, ~656.23, and 678.05 nm for **3B**, **4B**, **5B**, and **6B**, respectively. The electrons and holes distribute along the edges/corners. The percentage of Mo_4*d*_ in the electron is larger than that of S_3*p*_, while the percentage of S_3*p*_ in the hole is larger than that of Mo_4*d*_, leaving S atoms with the feature of hole and Mo atoms with the feature of the electron. In other words, the electron prefers to transfer from the S_3*p*_ to the connected Mo_4*d*_ along the edges/corners of **B**. The maximum absorption is ~502.21, ~654.45, ~633.81, and 845.93 nm for **3C**, **4C**, **5C**, and **6C**, respectively. The electron and hole distribute at the corners with the S atoms at the top vertex acting as hole and the Mo atoms neighboring acting as the electron (see Table 6 and Figure 6).

Overall, the S_0_→S_n_ transitions corresponding to the maximum absorption of the Mo-terminated triangle MoS_2_ clusters with 100% S coverage **A** come from two aspects, the *d*-*d* transitions of the Mo atom and transitions of S_3*p*_ along the edge. The S_0_→S_n_ transitions of **B** and **C** occur at the edges/corners, with an electron transferring from the S atoms to the neighboring Mo atoms. In other words, the S atoms act as the hole and the adjacent Mo atoms act as the electron in the case of the clusters with no dangling of edge S atoms in them. The S_0_→S_1_ transition directly leads to the lowest excited state and is always the excited state for luminescence. For the S_0_→S_1_ transition of **A**, the electron and hole mainly distribute throughout the whole triangular clusters; this can be observed faintly at the S atoms along the edges (see Figure 4). Referred to the data in Table 4, the percentage of Mo_4*d*_ in electron and hole not only takes a larger proportion but also has nearly the same values, which implies obvious *d*-*d* transitions of the Mo atoms. The S_0_→S_1_ transitions of **B** also occur at the edges/corners, as shown in Figure 5. By referring to the data in Table 5, both the Mo and S atoms possess the distribution of electrons and holes, which ascribes the S_0_→S_1_ from local transition. For the S_0_→S_1_ transitions of **C**, the percentage of Mo_4*d*_ in the electron is larger than that in the hole, while the percentage of S_3*p*_ in the hole is larger than that in the electron, implying significant electron transfer from the S atoms to the neighboring Mo atoms along the edges (see Figure 6). 

## 4. Conclusions

The electronic properties of three types of triangle MoS_2_ clusters, **A** (Mo-edge passivated with two S atoms), **B** (Mo-edge passivated with one S atom), and **C** (S-edge) have been explored using quantum chemistry methods in the present paper. The DOS appears to have different features in **A**, **B**, and **C**. For **A**, the MOs can be divided into two types: the edge state from S_3*p*_ along the edges, the hybrid state from Mo_4*d*_ and S_3*p*_ covering the whole cluster. Evident edge/corner states appear in the FMOs of **B** and **C**. The hybridization of Mo_4*d*_ and S_3*p*_ in **B** generate the occupied and unoccupied MOs. The occupied MOs mainly come from S_3*p*_, and the unoccupied MOs from the hybridization of Mo_4*d*_ and S_3*p*_ in **C**.

For the electron excitation processes of **A**, **B**, and **C**, the absorption peaks show a red shift along with increasing the size of the clusters. Electron–hole analysis indicated that the S_0_→S_1_ transitions appear to have different characteristics compared with the S_0_→S_n_ transitions (associated with the intense absorption in the electronic absorption spectrum) in **A**, **B**, and **C**. In **A**, the S_0_→S_1_ transitions mainly stem from the *d*-*d* transitions of the Mo atoms, while the S_0_→S_n_ transitions come from the *d*-*d* transitions of the Mo atom or transitions of the S_3*p*_ along the edges. Both the S_0_→S_1_ and S_0_→S_n_ transitions of **B** occur at the edges/corners, with the local S_0_→S_1_ transitions originating from Mo_4*d*_ and S_3*p*_, but charge transfer S_0_→S_n_ transitions caused by the electron transferring from S_3*p*_ to the connected Mo_4*d*_. In **C**, the S_0_→S_1_ transitions mainly occur along the edge with the S atoms acting as hole and the adjacent Mo atoms as electron, while the S_0_→S_n_ transitions mainly occur at the corners, where the two S atoms at the vertex acts as the hole and the adjacent Mo atoms act as the electron.

## Figures and Tables

**Table 1:** Formation energy *E*_formation_ (eV), energy of the highest occupied molecular orbital (HOMO) *E*_H_ (eV), energy of the lowest unoccupied molecular orbital (LUMO) *E*_L_ (eV), energy gap between HOMO and LUMO *E*_gap_ (eV), as well as orbital composition of HOMO *C*_H_ and LUMO *C*_L_ of **3A**, **4A**, **5A**, and **6A**.

	3A	4A	5A	6A
*E*_formation_	−151.84	−240.28	−348.85	−476.47
*E*_H_	−5.06	−5.55	−5.29	−5.71
*E*_L_	−6.84	−6.25	−5.95	−6.45
*E*_gap_	1.78	0.70	0.66	0.73
*C*_H_	S: *p*_x_, *p*_y_	S: *p*_x_, *p*_y_	Mo: *d*_xy_, *d*_x2y2_, *d*_z2_ S: p_x_, p_y_	S: *p*_x_, *p*_y_
*C*_L_	Mo: *d*_xy_, *d*_x2y2_ S: *p*_x_, *p*_y_	S: *p*_x_, *p*_y_	S: *p*_x_, *p*_y_	Mo: *d*_xy_, *d*_x2y2_, *d*_z2_ S: *p*_x_, *p*_z_

**Table 2:** Formation energy *E*_formation_ (eV), energy of HOMO *E*_H_ (eV), energy of LUMO *E*_L_ (eV), energy gap between HOMO and LUMO *E*_gap_ (eV), as well as orbital composition of HOMO *C*_H_ and LUMO *C*_L_ of **3B**, **4B**, **5B**, and **6B**.

	3B	4B	5B	6B
*E*_formation_	−120.27	−200.28	−297.42	−415.25
*E*_H_	−5.80	−5.23	−5.72	−5.96
*E*_L_	−7.00	−7.33	−6.74	−7.07
*E*_gap_	1.19	2.10	1.02	1.11
*C*_H_	Mo: *d*_x2y2_, *d*_z2_, *d*_xz_ S: *p*_x_, *p*_y_, *p*_z_	Mo: *d*_z2_ S: *p*_x_, *p*_y_, *p*_z_	Mo: *d*_yz_, *d*_x2y2_, *d*_z2_ S: *p*_x_, *p*_y_, *p*_z_	Mo: *d*_xy_, *d*_z2_ S: *p*_y_, *p*_z_
*C*_L_	Mo: *d*_xy_, *d*_x2y2_, *d*_z2_ S: *p*_x_, *p*_y_, *p*_z_	Mo: *d*_xz_, *d*_yz_ S: *p*_x_, *p*_y_	Mo: *d*_xy_, *d*_x2y2_, *d*_z2_ S: *p*_x_, *p*_y_	Mo: *d*_xy_, *d*_x2y2_, *d*_z2_ S: *p*_x_, *p*_y_, *p*_z_

**Table 3:** Formation energy *E*_formation_ (eV), energy of HOMO *E*_H_ (eV), energy of LUMO *E*_L_ (eV), energy gap between HOMO and LUMO *E*_gap_ (eV), as well as orbital composition of HOMO *C*_H_ and LUMO *C*_L_ of **3C**, **4C**, **5C**, and **6C**.

	3C	4C	5C	6C
*E*_formation_	−137.42	−219.60	−321.09	−441.37
*E*_H_	−5.59	−5.74	−5.57	−5.71
*E*_L_	−6.54	−6.67	−6.74	−6.79
*E*_gap_	0.96	0.92	1.17	1.08
*C*_H_	S: *p*_x_	S: *p*_x_, *p*_z_	S: *p*_x_, *p*_y_, *p*_z_	S: *p*_x_, *p*_z_
*C*_L_	Mo: *d*_xy_, *d*_x2y2_, *d*_z2_ S: *p*_y_, *p*_z_	Mo: *d*_x2y2_, *d*_z2_, *d*_xz_ S: *p*_x_, *p*_y_, *p*_z_	Mo: *d*_xy_, *d*_x2y2_, *d*_z2_ S: *p*_x_, *p*_y_, *p*_z_	Mo: *d*_x2y2_, *d*_z2_ S: *p*_x_, *p*_y_, *p*_z_

**Table 4:** Transition between the ground state and the lowest excited state S_0_→S_1_, transition between the ground state and the state with the largest oscillator strength S_0_→S_n_ (normally associate with the intense absorption in the electronic absorption spectrum, n is usually different in different clusters), and the percentage of Mo_4*d*_ and S_3*p*_ in electron and hole of **3A**, **4A**, **5A**, and **6A**.

		Electron, %	Hole, %
**3A**	S_0_→S_1_	Mo*_4d_*: 60.29 S_3*p*_: 24.17	Mo*_4d_*: 72.76 S_3*p*_: 9.36
	S_0_→S_21_	Mo*_4d_*: 16.97 S_3*p*_: 64.86	Mo*_4d_*: 17.85 S_3*p*_: 62.80
**4A**	S_0_→S_1_	Mo*_4d_*: 64.27 S_3*p*_: 20.03	Mo*_4d_*: 60.14 S_3*p*_: 15.68
	S_0_→S_48_	Mo*_4d_*: 41.28 S_3*p*_: 28.71	Mo*_4d_*: 47.34 S_3*p*_: 26.80
**5A**	S_0_→S_1_	Mo*_4d_*: 72.11 S_3*p*_: 8.84	Mo*_4d_*: 70.92 S_3*p*_: 6.44
	S_0_→S_36_	Mo*_4d_*: 45.32 S_3*p*_: 23.84	Mo*_4d_*: 50.55 S_3*p*_: 12.13
**6A**	S_0_→S_1_	Mo*_4d_*: 65.27 S_3*p*_: 0	Mo*_4d_*: 60.44 S_3*p*_: 0
	S_0_→S_27_	Mo*_4d_*: 37.62 S_3*p*_: 31.75	Mo*_4d_*: 29.56 S_3*p*_: 35.33

**Table 5:** S_0_→S_1_ transition, S_0_→S_n_ transition, and the percentage of Mo_4*d*_ and S_3*p*_ in electron and hole of **3B**, **4B**, **5B**, and **6B**.

		Electron, %	Hole, %
**3B**	S_0_→S_1_	Mo*_4d_*: 33.09 S_3*p*_: 56.59	Mo*_4d_*: 48.57 S_3*p*_: 39.28
	S_0_→S_73_	Mo*_4d_*: 53.17 S_3*p*_: 31.18	Mo*_4d_*: 31.98 S_3*p*_: 45.78
**4B**	S_0_→S_1_	Mo*_4d_*: 65.12 S_3*p*_: 18.77	Mo*_4d_*: 51.49 S_3*p*_: 23.19
	S_0_→S_17_	Mo*_4d_*: 53.68 S_3*p*_: 23.46	Mo*_4d_*: 6.98 S_3*p*_: 64.08
**5B**	S_0_→S_1_	Mo*_4d_*: 52.29 S_3*p*_: 32.33	Mo*_4d_*: 56.36 S_3*p*_: 24.52
	S_0_→S_25_	Mo*_4d_*: 49.56 S_3*p*_: 15.81	Mo*_4d_*: 12.53 S_3*p*_: 57.54
**6B**	S_0_→S_1_	Mo*_4d_*: 50.53 S_3*p*_: 31.77	Mo*_4d_*: 45.67 S_3*p*_: 26.18
	S_0_→S_20_	Mo*_4d_*: 57.88 S_3*p*_: 16.71	Mo*_4d_*: 6.52 S_3*p*_: 66.06

**Table 6:** S_0_→S_1_ transition, S_0_→S_n_ transition, and the percentage of Mo_4*d*_ and S_3*p*_ in electron and hole of **3C**, **4C**, **5C**, and **6C**.

	Transition	Electron, %	Hole, %
**3C**	S_0_→S_1_	Mo_4*d*_: 58.26 S_3*p*_: 27.97	Mo_4*d*_: 20.09 S_3*p*_: 64.86
	S_0_→S_17_	Mo_4*d*_: 55.81 S_3*p*_: 26.89	Mo_4*d*_: 34.75 S_3*p*_: 43.73
**4C**	S_0_→S_1_	Mo_4*d*_: 47.55 S_3*p*_: 36.23	Mo_4*d*_: 2.55 S_3*p*_: 86.51
	S_0_→S_31_	Mo_4*d*_: 54.91 S_3*p*_: 41.67	Mo_4*d*_: 21.46 S_3*p*_: 74.25
**5C**	S_0_→S_1_	Mo_4*d*_: 57.86 S_3*p*_: 23.43	Mo_4*d*_: 2.79 S_3*p*_: 83.63
	S_0_→S_42_	Mo_4*d*_: 49.91 S_3*p*_: 31.92	Mo_4*d*_: 28.54 S_3*p*_: 49.41
**6C**	S_0_→S_1_	Mo_4*d*_: 55.02 S_3*p*_: 17.42	Mo_4*d*_: 2.45 S_3*p*_: 76.37
	S_0_→S_19_	Mo_4*d*_: 42.57 S_3*p*_: 21.73	Mo_4*d*_: 12.33 S_3*p*_: 53.19

## Data Availability

Not applicable.
